# Age, period and cohort analysis of suicide trends in Australia, 1907–2020

**DOI:** 10.1016/j.lanwpc.2024.101171

**Published:** 2024-08-13

**Authors:** Matthew J. Spittal, Rachel Mitchell, Angela Clapperton, Adrian Laughlin, Mark Sinyor, Andrew Page

**Affiliations:** a Melbourne School of Population and Global Health, The University of Melbourne, Melbourne, Australia; b Department of Psychiatry, Sunnybrook Health Sciences Centre, Toronto, Canada; c Department of Psychiatry, University of Toronto, Toronto, Canada; d Department of General Practice and Primary Care, The University of Melbourne, Melbourne, Australia; e Translational Health Research Institute, Western Sydney University, Penrith, Australia

**Keywords:** Suicide, Age-period-cohort, Australia, Suicide trends

## Abstract

**Background:**

Suicide rates have been increasing in Australia since the mid-2000s, especially for women aged ≤25 years. We conducted an age-period-cohort study to investigate these recent trends in the context of historical Australian suicide rates.

**Methods:**

Data on annual suicides in Australia from 1907 to 2020 were extracted from the General Record of Incidence of Mortality. We modelled age-specific effects for a reference cohort, after adjustment for period effects.

**Findings:**

We found evidence of age, cohort and period effects. For males, compared to the cohort born in 1946–1950, rates were higher for all cohorts born after this year. The period effect showed peaks in the risk of male suicide in the mid 1960s and the early 1990s, followed by a decline in risk until early 2010, after which the risk began to rise again. For females, compared to the cohort born in 1946–1950, the risk of suicide was higher for all cohorts born after this, with the highest risk for those born in 2006–2010. The period effect for females showed an elevated risk of suicide in the mid 1960s followed by a sharp decline, and an increase in risk after 2009.

**Interpretation:**

Suicide rates in Australia have fluctuated substantially over time and appear to be related to age trends as well as period and cohort trends. Advocacy and policy making tends to focus on contemporaneous changes in suicide rates. However, this study shows that focusing only on year-on-year changes in suicide rates ignores underlying trends for specific population birth-cohorts.

**Funding:**

None.

**Research in context**

**Evidence before this study**

Previous research has shown that population-level changes in suicide rates may be due to a combination of the age trends, temporal trends and birth cohort trends, with the relative contribution of each differing across settings. We searched PubMed titles and abstracts with terms “(suicide) AND (age-period-cohort) AND (Australia)”, with no language restrictions, from database inception to 21 February 2024. We identified four studies of long-term suicide trends in Australia that examined trends from the perspective of age, period and birth cohort effects. One study, conducted in New South Wales using suicide data from 1868 to 1998, found no evidence of cohort effects amongst men or women and concluded that changes in rates were due predominately to temporal trends affecting everybody (period effects). Two studies were conducted using national suicide data, one 1919–1999 and the other 1907–2010. The first study concluded that changes in suicide trends were due to period effects and not cohort effects. The second study concluded that both period and cohort effects were present: cohorts born after 1970–1974 had an elevated lifetime risk of suicide and cohorts who experienced high unemployment during the 1990s continued to carry high risk of suicide as they aged. We also identified a study of youth suicide (those aged 15–24 years) conducted over the 1964–1997 period which concluded that changes in youth suicides was largely attributable to temporal trends, not birth cohort trends. All studies acknowledged that their inability to resolve the identification problem—the problem of not being able to simultaneously account for age, period and cohort effects—as a major limitation.

**Added value of this study**

We used the General Record of Incidence of Mortality workbooks for suicide (1907–2020) to plot age-standardised rates for men and women over time and to simultaneously analyse age, period and cohort effects by sex. We report how trends in suicide vary over time, with peaks in suicide rates for both sexes corresponding to the Great Depression of the 1930s, the availability of sedatives in the 1960s and the onset of the Global Financial Crisis in the late 2000s. For men, we additionally observed an increase in suicides that corresponded with high unemployment in the 1990s. For both sexes, we report the association between age and suicide as rates per 100,000, and we report how these rates increase or decrease by birth cohort and time period. We identify clear cohort effects for men and women, with the lifetime risk of suicide increasing for each birth cohort born after 1946–1950. For women, this trend has continued unabated; for men, the cohort trend peaked in the early 2000s.

**Implications of all the available evidence**

Advocacy and policy making tends to focus on year-on-year changes in the suicide rates. Yet, suicide rates in Australia have fluctuated substantially over time and appear to be related to age trends as well as period and cohort trends. Optimal public health approaches to prevention would consider underlying trends for specific birth-cohorts in the population.

## Introduction

In Australia, an increase in suicide rates among young people—especially young women—has been observed in recent national mortality statistics.^1^ One study found that suicide rates among young women (10–24 years) increased by 3% per year between 2004 and 2014.^2^ Similar patterns have also been observed in several other high-income countries over comparable time periods.^3^, ^4^, ^5^, ^6^ This has occurred against a backdrop of worsening mental health in Australia and elsewhere, particularly for among those born in the 1990s,^7^ and also a period of widening socio-economic inequality and the emergence of smartphones and rapid increases in digital media use.

One way of viewing long-term suicide trends is to examine changes from the perspective of age, period and cohort effects. An age effect refers to variation in suicide risk that is linked to aging, for example because of biological or social processes. A period effect refers to variation due to a common cause that effects the whole population at the same time, regardless of age. A cohort effect refers to variation due to birth cohort (i.e., the year of birth) and implies that some cohorts carry a greater lifetime risk of suicide than others. Examples of how long-term suicide trends have changed in response to these three effects include the role of unemployment and economic change in the male ‘youth suicide epidemic’ during the 1980s and 1990s^8^^,^^9^; the impact of sedative availability on female suicide during the 1960s^10^; the impact of economic crisis such as the Great Depression^11^^,^^12^ and the more recent Global Financial Crises.^13^, ^14^, ^15^ Also of importance have been changes in workforce factors relating to female labour force participation,^16^ and changes in relationship breakdown rates.^17^^,^^18^ Less well understood, but potentially relevant for younger birth cohorts, is gender differences in the consumption of digital media, and the relationship between heavy digital media use and having one or more risk factors for suicide (especially among young women).^19^ Our aim, therefore, was to investigate the role of age, period and cohort effects on long-term suicide rates in Australia for males and females from 1907 onwards.

## Methods

### Data source

Ethical approval for this study was obtained from the University of Melbourne’s Low and Negligible Risk Committee (ID 2023-27242-41349-3). We obtained annual counts of suicides from 1907 to 2020 by age groups and sex from the Australian Institute of Health and Welfare (AIHW). This information is contained within the General Record of the Incidence of Mortality (GRIM) books and is freely available to download from AIHW’s website.^20^ Suicides were identified using the World Health Organization’s International Statistical Classification of Diseases and Related Health Problems (ICD). ICD-1 coding was used for the 1907–1917 period, ICD-2 for 1918–1921, ICD-3 for 1922–1930, ICD-4 for 1931–1939, ICD-5 for 1940–1949, ICD-6 for 1950–1957, ICD-7 for 1958–1967, ICD-8 for 1968–1978, ICD-9 for 1979–1996 and ICD-10 for 1997 onwards. In the GRIM books, age is coded in 5-year bands, from 0–4 to ≥85 years. The 5-year age bands, combined with the yearly data, meant birth cohorts could be classified into 5-year bands, from 1818–1822 to 2016–2020 and increasing in yearly intervals (1818–1822, 1819–1823, and so on). Sex was coded into two categories, males and females. GRIM books also record the estimated resident population size for each year, age and sex groups.

### Inclusion criteria

We included all suicides between 1907 and 2020 in people aged 10–84 years. Suicides outside these age ranges were excluded. Those aged <10 years were excluded as there were fewer than 10 suicides in the 0–4 and 5–9 age groups over the entire study period, making any estimates derived from these groups unstable. Those aged ≥85 years were excluded as this was the upper age-band in the original data, thus once individuals were in this category, we could no longer identify their cohort as they continued to age (for example, a person aged 85 year would be assigned to the same birth cohort as person aged 95 years). These criteria resulted in birth cohorts that ranged from 1823–1827 to 2006–2010.

### Descriptive analyses

We first report age-standardised suicide rates over time for males and females. Rates were calculated using direct standardisation to the current Australian standard population (the estimated resident population in 2001) aged 10–84 years. For each sex, we then report rates over time by age (with age grouped in 15-year bands from 10–24 to 70–84) and age-specific rates by birth cohort (with the midpoint of each birth cohort grouped into decades). Rates within age or cohort groups were smoothed using a moving average filter (a rolling mean calculated using two lagged observations, the current observation and two lead observations).

### Age, period and cohort analyses

The challenge when distinguishing between age, period and cohort effects is the identifiability problem due to year of birth, which defines the cohort, being linearly related to age in any given period. That is, Cohort = Period – Age. To disentangle the independent effects of these three components, we therefore used an approach developed by Carstensen^21^ which allows all three components to be estimated in a single model. Briefly, age, period and cohort are entered into a model as continuous variables using restricted cubic splines to model any non-linear patterns. To overcome the identifiability problem, a linear temporal change, or drift parameter, is applied to either the cohort function or the period function after detrending the cohort and period spline variables. When the drift parameter is applied to the cohort function (referred to as an APC model), the model estimates age-specific rates for a reference period after adjustment for cohort effects. When the drift parameter is applied to the period function (referred to as an ACP model), the model estimates age-specific rates for a reference cohort after adjustment for period effects. Models that exclude either period or cohort terms can also be fit to the data within this framework (referred to as AC and AP models).

As our primary interest was in estimating the risk of suicide by age and how this differs across time periods and cohorts, we began by estimating an ACP model and comparing this to an AC model (age and cohort effects) and an AP model (age and period effects) for males and females separately. We did this using the apcfit command that implements Carstensen’s approach in Stata. We converted the GRIM books for suicides into an analytic dataset organised such that each row of data represented the number of suicides and the person time by each category of sex (male, female), age (5-year bands), period and cohort. After stratifying by sex, we undertook exploratory analyses to identify both the number of knots for the cohort and period functions that fit the data best as well as the best fitting model (ACP, AC, AP). Model fit was determined using goodness of fit statistics (Akaike information criterion and Bayesian information criterion). All models were estimated using the generalised linear model with a log link. We then plotted the results of the best fitting model for males and females, reporting age-specific effects as a rate (per 100,000 person years) and cohort and period effects as rate ratios. In this parameterisation, because the drift term was applied to the cohort function, the rate ratios for the cohort effects are interpreted as relative to the reference cohort after adjustment for age and period effects. The rate ratios for the period effect represent the residual effect after adjustment for the age and cohort prediction and were calculated relative to the reference period.^22^ We used those born the in the 1946–1950 period as the reference cohort (for comparability with other studies) and 1980 as the reference period as this was prior to sustained economic reform in Australia in the 1980s.^23^ To assess model fit, we calculated the predicted rates from the final models and compared these to the observed rates for a sample of birth cohorts (cohorts 10 years apart from the 1911–1915 birth cohort to the 2001–2005 birth cohort). All analyses were undertaken in Stata version 18.0.

### Role of the funding source

There was no funding source for this study.

## Results

### Descriptive results

**Fig. 1:**
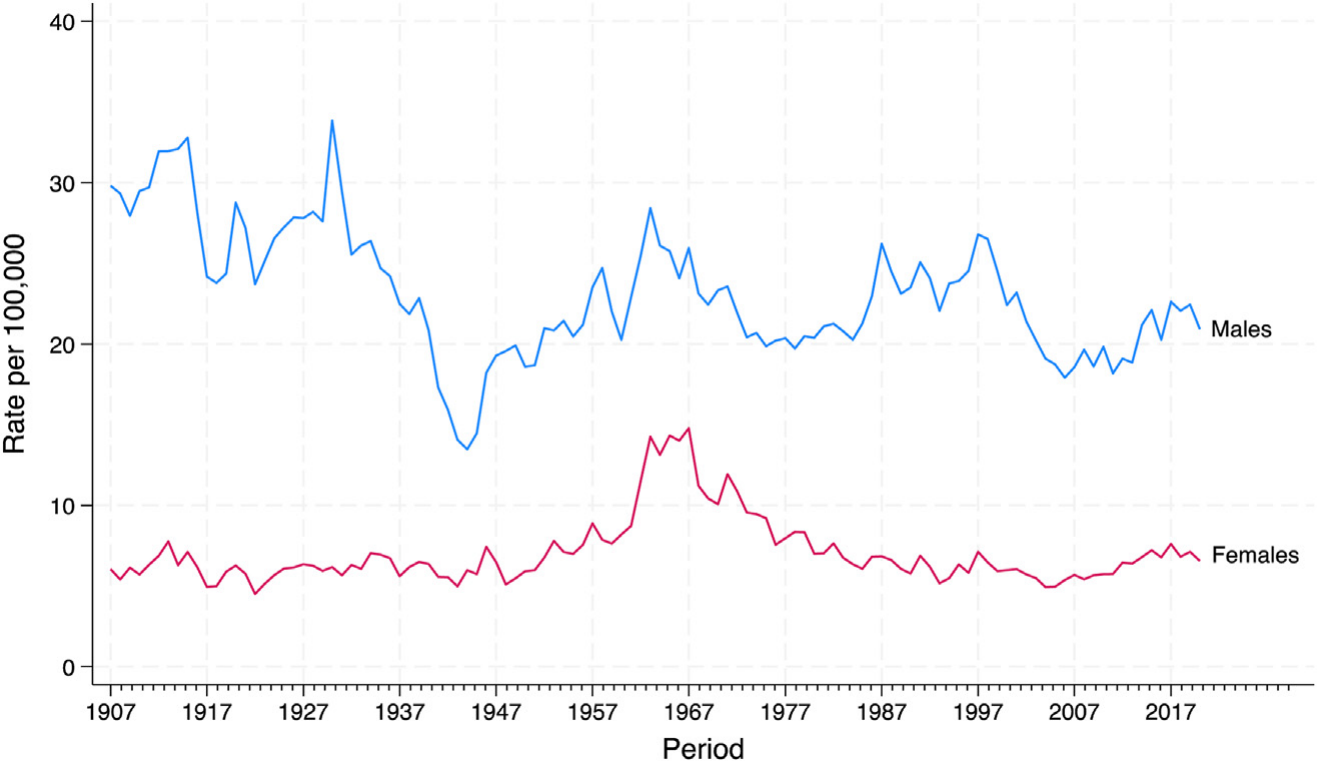
Age-standardised suicide rates, 1907–2020. Note. Direct age-standardisation to the 2001 Australian population aged 10–84 years of age.

During the study period, there were a total of 164,371 suicides, 76% of these in males and 24% in females. Fig. 1 shows the age-standardised suicide rates (per 100,000 person years) for males and females. Male rates were substantially higher than female rates across the entire period of study. There was a notable drop in male suicide rates in the early 1940s and an increase in female suicides beginning in the early 1960s and continuing until the mid 1970s. In the recent period, suicide rates were at their lowest for both sexes in the mid 2000s and appear to have increased since then.

**Fig. 2:**
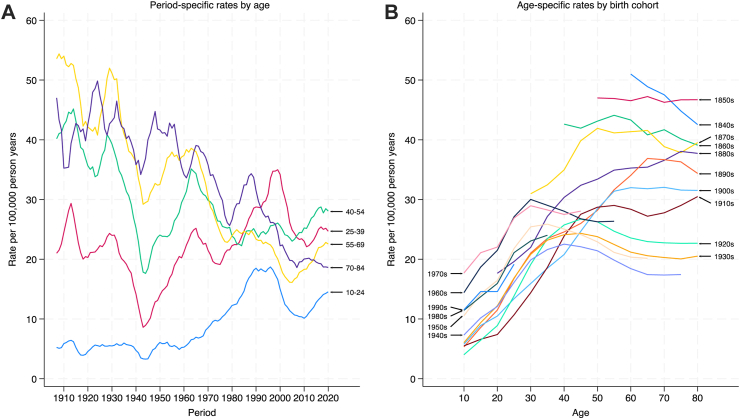
Trends in the risk of suicide over time by age (left panel) and trends in the risk of suicide over age by cohort (right panel), males.

Fig. 2 (**panel A**) shows the smoothed rates over time by age groups for males. In general terms, the suicide rates among males aged 40–54, 55–69 and 70–84 declined over time while rates in the 10–24 and 25–39 age groups increased. There were notable declines in the 1940s for all age groups, peaks in the 1960s for all age groups except the youngest group, a peak in the 1990s for 10–24 age group and again in the 2000s for the 25–39 age group. **Panel B** shows smoothed rates over the lifetime by birth cohort. Overall, suicide rates for men increase with age. But there is considerable variation by birth cohort. Some cohorts, for instance the 1840–1900 birth cohorts have much higher rates in older age then cohorts born after this, for instance the 1920 and 1930 cohorts. Cohorts born after the 1960s have higher rates in young age then those born earlier (e.g., the 1920 and 1930 cohorts).

**Fig. 3:**
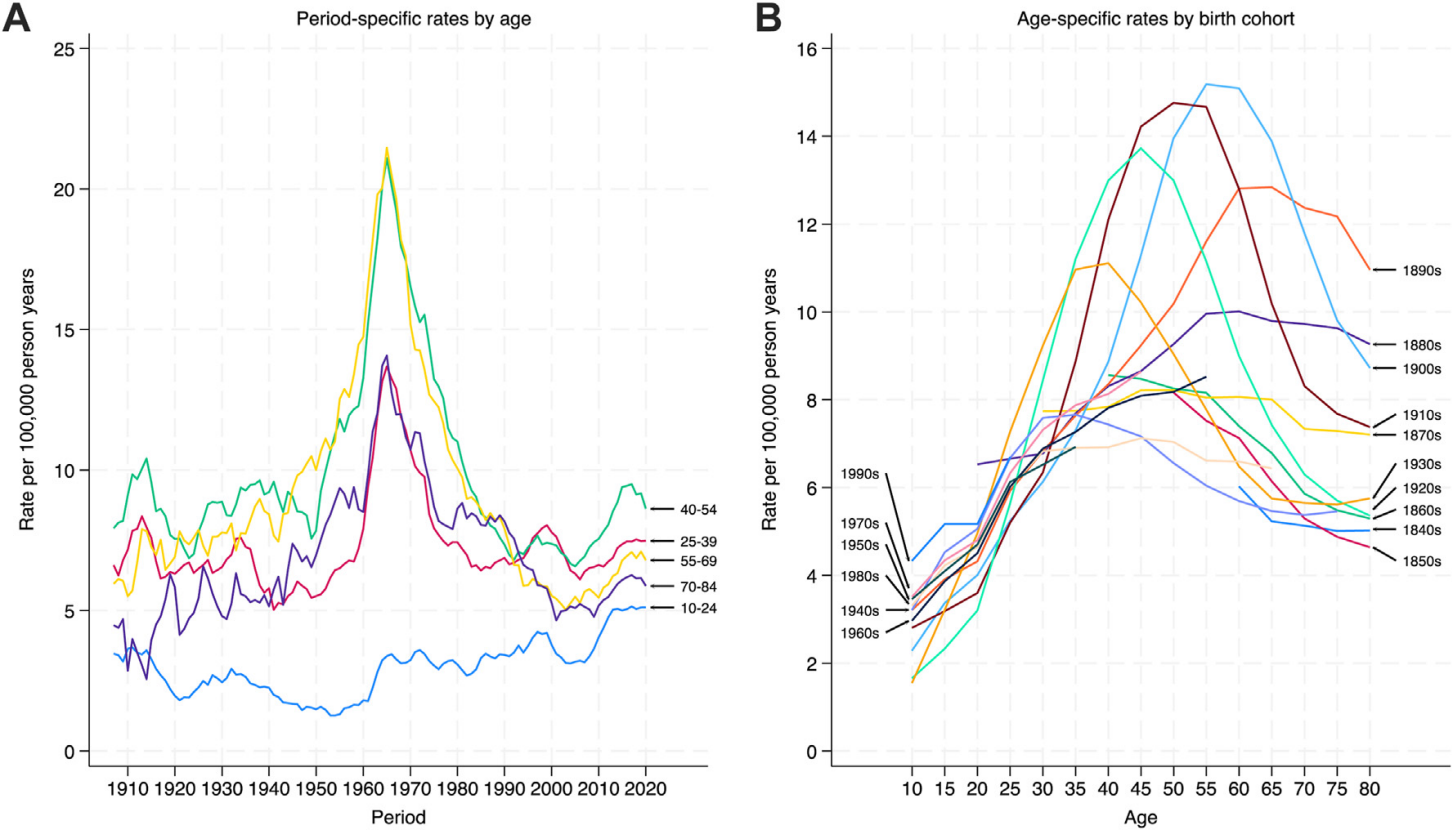
Trends in the risk of suicide over time by age (left panel) and trends in the risk of suicide over age by cohort (right panel), females.

For females, the smoothed rates over time indicate a clear peak in suicides in the 1960s for all groups except those aged 10–24 (Fig. 3, panel A). The two age groups with the highest rates over most of the period were the 40–54 and 55–69 groups. Rates in the 10–24 age group have been steadily rising since the 1960s, albeit with a brief decline during the 2000–2010 period. The plot of suicide rates over the lifetime (**panel B**) show a pattern of suicide rates increasing with age and peaking between ages 45 and 60. Rates are higher for some birth cohorts than others, for instance the previously described peak was highest in those born in the 1860s, 1890s, 1900s and 1910s. Rates in early age appear higher in those born in the 1990s than in those born in other periods.

### Age, period and cohort results

**Fig. 4:**
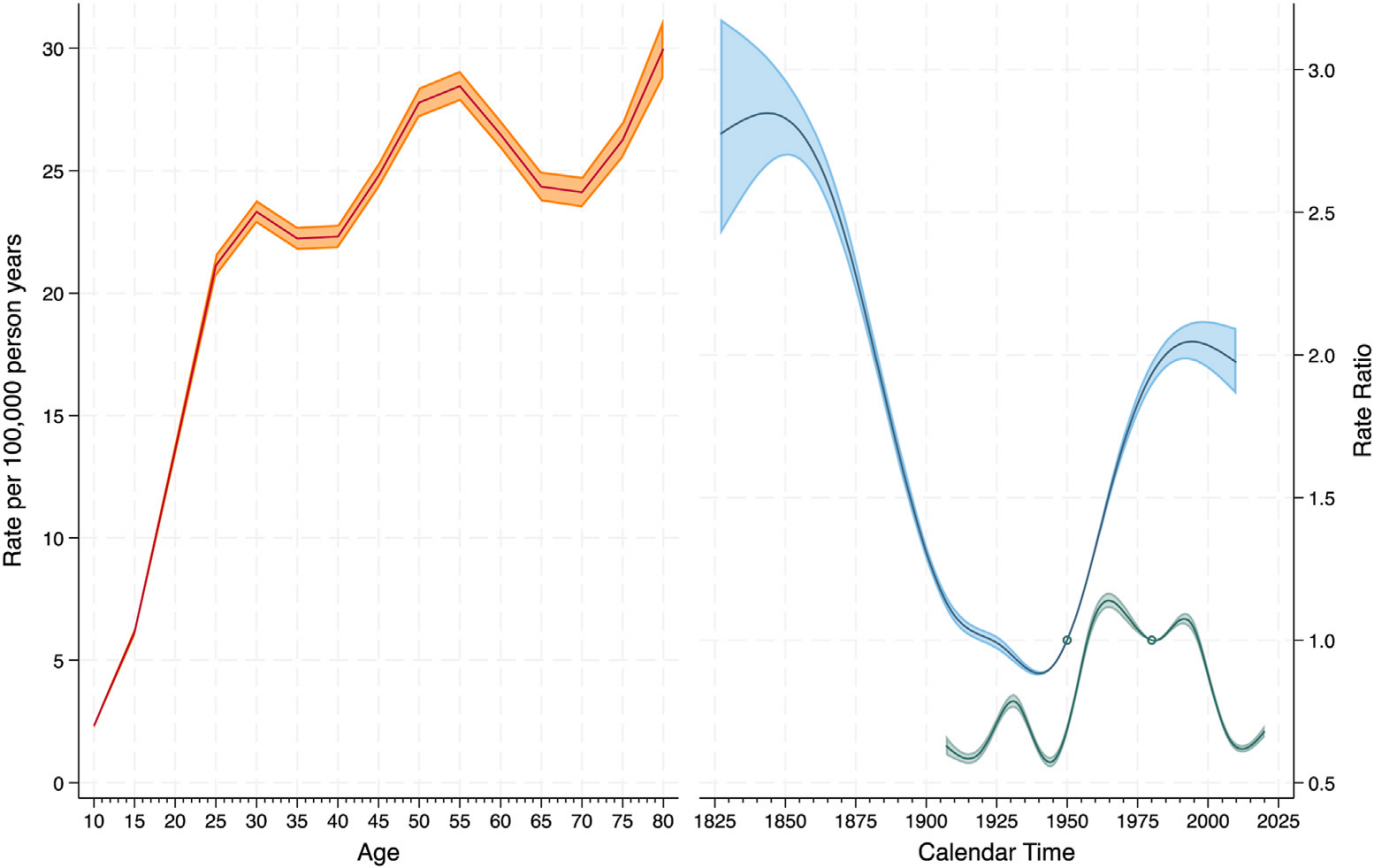
Estimates for age, cohort and period effects for male suicide rates (1907–2022) and 95% confidence intervals. Notes. The reference cohort is 1946–1950 birth cohort and the reference period is 1980. The estimated age-specific rates (left panel, in orange) are for this cohort and period. The rate ratio for cohort (right panel, in blue) estimates how the age-specific rates change relative to the 1946–1950 cohort. The rate ratio for period (right panel, in green) represents the residual period effects relative to the rate in 1980.

The best fitting model for males was the ACP model (age-specific rates for each cohort after adjustment for period effects) with 7 knots for the cohort function and 9 knots for the period function (See supplement, Supplementary Table S1 for goodness of fit statistics for all models and Supplementary Table S2 for coefficients for the best fitting model.). The estimated age-specific rates and the rate ratios (RR) derived from the best fitting model for males are shown in Fig. 4. The left-hand panel shows the estimated age-specific rates for males in the 1946–1950 birth cohort (the reference cohort) in the year 1980 (the reference period). For this cohort and period, male suicide rates were lowest at 10–14 years of age (rate = 2.3 per 100,000 person years), with peaks in the 30–34 age group (rate = 23.3 per 100,000 person years), 55–59 age group (rate = 28.4 per 100,000 person years) and 80–84 age group (30.0 per 100,000 person years). The right-hand panel shows how the age-specific rates (and their 95% confidence intervals) on the left increase or decrease relative to the reference cohort and period. The RRs for the cohort effect broadly had a U-shaped pattern. The age-specific suicide rates for those males born in the 1828–1832 cohort were 2.8 times higher than that of those born in the 1946–1950 cohort. For those born in 1936–1940 cohort (the nadir), rates were 0.88 times lower. The RRs subsequently increased for each cohort, for instance, for those males born in the 1996–2000 cohort, the rates were 2.03 times higher than they were for those born in the 1946–1950 cohort. There was some evidence of a slight decline in RRs for males born since 2000 (e.g., for the 2006–2010 cohort, the RR was 1.97). The residual RRs for the period effect demonstrate how the risk of suicide has changed over time. Relative to the risk of suicide in the 1980 period, there was an initial peak in the early 1930s (RR = 0.79) then a decline in risk each year until the mid 1940s (RR = 0.57). The risk of suicide than increased each year until a peak in the mid 1960s (RR = 1.14) followed by another decline that reached its nadir in the early 1980s (RR = 1.00). The risk then increased again, peaking in early 1990s (RR = 1.07) before declining sharply until the early 2010s (RR = 0.62). Since then, suicide rates for males have been increasing each year, with RR = 0.68 in 2020.

**Fig. 5:**
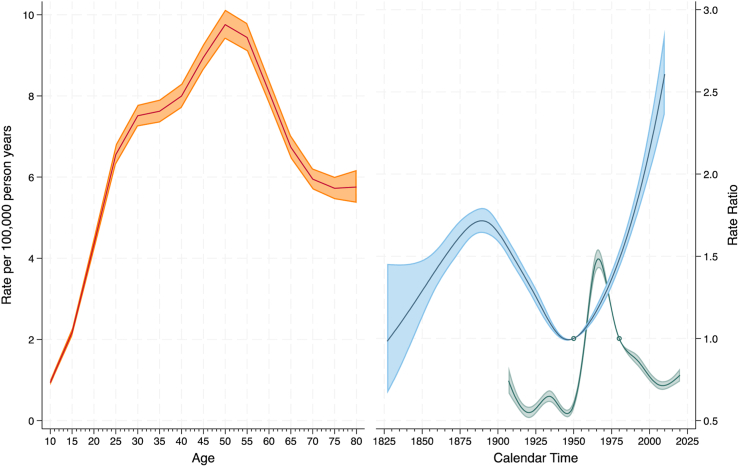
Estimates for age, cohort and period effects for female suicide rates (1907–2022) and 95% confidence intervals. Notes. The reference cohort is 1946–1950 birth cohort and the reference period is 1980. The estimated age-specific rates (left panel, in orange) are for this cohort and period. The rate ratio for cohort (right panel, in blue) estimates how the age-specific rates change relative to the 1946–1950 cohort. The rate ratio for period (right panel, in green) represents the residual period effects relative to the rate in 1980.

For females, the model that provided the best fit to the data was the ACP model with 7 knots for the cohort function and 8 knots for the period (See supplement, Supplementary Tables S3 and S4 for goodness of fit statistics for all models and model coefficients for the best fitting model). The age, period and cohort functions derived from the best fitting model for females are shown in Fig. 5. For the reference birth cohort and period, the estimated age-specific rates for females increased from 0.9 per 100,000 person years at age 10–14 to 9.8 per 100,000 person years at age 50–54. Rates then declined in older age to 5.7 per 100,000 person years in the 75–79 group. A complex pattern emerged for the cohort effects. The risk of suicide initially increased for each cohort born after 1823–1827, reaching a local maximum for the 1885–1889 birth cohort (from RR = 0.98 to RR = 1.70, both relative to the 1946–1950 birth cohort). The risk of suicide then declined for each cohort, with the minimum occurring for the 1943–1947 birth cohort (RR = 0.99). Subsequent to this, the risk of suicide has increased for each cohort born after this. The risk was highest for females born in the 2006–2010 birth cohort who had a risk of suicide that is 2.61 times that of females born in the 1946–1950 cohort. Similarly, the residual RRs for the period effect showed a complex pattern. Relative to the 1980 reference period, the risk of suicide declined each year between 1907 and 1920 (from RR = 0.74 to RR = 0.55). The risk of suicide then began to rise, reaching a local maximum in the mid 1930s (RR = 0.65) and then declining again until the mid 1940s (RR = 0.54). The risk of suicide then increased each year until the mid 1960s (RR = 1.48) followed by a decline in risk until 2009 (RR = 0.71) after which the risk of suicide began to increase again (RR = 0.78 in 2020).

A comparison of the observed and predicted rates (per 100,000 person years) for birth cohorts 10 years apart, from 1831–1835 to 2001–2005, showed close concordance for males and females (see supplement, Supplementary Figs. S1 and S2), supporting the validity of the models.

## Discussion

In this study of suicide in Australia between 1907 and 2020, we found evidence of age effects (risk of suicide changing as people age), cohort effects (some birth cohorts carrying a greater lifetime risk of suicide than others) and period effects (periods where suicide rates were elevated for all age groups). These effects were observed in males and females. Recent data has suggested an increased risk of suicide among young women.^2^ The long-term data we analysed showed that each cohort of women born after 1946–1950 has a higher risk of suicide than the cohort born previously. Our data also showed that the suicide risk for all women has increased since the late 2000s. This suggests that the recently observed increase in suicide among young women may be a function of both a cohort effect and a recently rising period effect. Should these two trends continue at the same rate, the suicide rate among women is expected to continue to increase. In men, we also found evidence of a cohort effect and a period effect, although these recent trends appeared to be counteracting one another. Like women, the risk of suicide in men has increase for each cohort born after 1946–1950, although there is some evidence that this trend may have begun to decline for those cohorts born after 2000. The period effect showed sustained reductions in the overall male suicide rates since the 1970s with a recent increase in the risk of suicide since the late 2000s. The combined effect of these two trends on future male suicide rates is difficult to judge and will depend on the relative importance of each.

The findings were observed in Australia appear to be consistent with other countries. Recent age-period-cohort studies of suicide have been conducted in Brazil,^24^ Canada,^25^ China,^26^ England and Wales^27^ Hong Kong,^28^ Japan,^29^ Russia,^30^ South Korea,^31^ Spain,^32^ Switzerland,^33^ Taiwan^28^ and the United States,^34^ While these studies have used a variety of different methods to account for the identification problem inherent in age-period-cohort studies, common findings have emerged, especially in relation to age and cohort effects. Most countries show a similar age trends for men and women to Australia, notably the United States, Canada, Brazil, England and Wales and Switzerland. Trends for males (but not females) are also similar in Hong Kong and Taiwan, South Korea and Spain. Age trends for both sexes in Russia appear to be different to Australia. With respect to cohort effects, except for China and Russia, most countries show the risk of male suicide rising for each cohort born since the early 1950s. In China and Russia, the lifetime risk of suicide for males has declined in each subsequent cohort. The cohort effects for women is less clear. Female suicide rates increased for the cohorts born after the early 1980s in Canada^25^ and Japan and after the mid-1960s in South Korea.^29^ In China and Russia, the risk has declined for each cohort. In Hong Kong and Taiwan, the there was no evidence of a cohort effect for women, with rates relatively stable from one cohort to the next in each country.^28^

Our study does not identify the mechanisms that may be underlying the cohort and period effects. Regarding cohort effects, which were similar for males and females, several explanations have been proposed. Later born cohorts in Australia (post the 1970s and 1980s) have experienced an epoch of economic deregulation resulting in higher under-employment and casualised work, and previous research has suggested a correlation between cohort-specific under-employment and cohort-specific suicide.^9^ Inadequate work has been shown to be associated with poor self-rated health,^35^ and depressive symptoms,^36^ and socio-economic and employment factors are also determinants of more proximal factors for suicide such as mental health conditions and psychological distress^37^ and precipitating factors for suicide such as exposure to interpersonal conflict,^38^ relationship breakdown^39^ and drug and alcohol use.^40^ During more recent periods in Australia, corresponding with these younger-age cohorts, there has been increases in self-reported depression and self-harm,^41^ and decreases in vocational participation^42^ and increases in more lethal methods among young women (particularly those aged 10–14 years).^2^ The period effects we observed were broadly similar for each sex. We observed a peak in suicides in the early 1930s among men and in the mid 1930s among women. In both sexes, we observed declines in suicides in the mid 1940s and large subsequent increases that peaked in the mid 1960s. There were some differences between the sexes after this. In males, there was a third peak in the early 1990s, but this did not occur among women. Finally, there is evidence that suicide rates have begun to rise for both sexes since the late 2000s. These patterns have previously been attributed to the Great Depression in the 1930s,^43^ the artefactual decline in suicide during World War II due to servicemen being excluded from mortality statistics,^44^ the increased availability of sedatives during the 1960s^10^ and the male ‘youth suicide epidemic’ in the 1980s and 1990s.^45^ The recent increases in suicides observed in both sexes since the late 2000s may be due, in part, to the Global Financial Crisis that begun in 2007.^46^

Our data show that in young women, the period effect could be compounding the cohort effect, whereas in young men, it could be offsetting it. While there are several possible explanations for this, the most obvious is the growing consumption of digital media that began in the early-to-mid 2000s and differences in the way young women and men interact with this media.^19^ Large and representative surveys (≥220,000 participants) from the US and the UK show that young women spend more time using smartphones than young men, and spend more time on social media and texting. Young men spend more time gaming. The evidence also suggests that some types of digital media consumption are more harmful than others, for instance social media is associated with depressive symptoms in young women but not young men^47^ whereas there is emerging evidence that gaming may be protective against mental health problems.^48^ (This is separate to the issue of compulsive gaming, which does appear to be harmful, but is of much lower prevalence in the general population.^49^) A sizeable body of evidence has linked both screen time and social media use to depressive symptoms in young people.^47^^,^^50^^,^^51^ Heavy electronic device use (≥5 h per day) has been associated with the presence of one or more risk factors for suicide (particularly for young women). In addition to the frequency of digital media use, there is evidence that the content of the digital media may be harmful to young women. One example is the prominent normalization of self-harm behaviour in social media^52^ which may promote contagion, especially among young women.^53^ Another example is the glamourisation of suicide and self-harm by female protagonists in online streaming services. The Netflix series *13 Reasons Why* presented sensationalised and repetitive suicide-related content over its first season, and this was subsequently shown to be associated with increases in suicide and suicide-related behaviour, notably among young women.^54^, ^55^, ^56^ Taken together, these findings imply that the harmful aspects of digital media use have had a more pronounced effect on young women in the general population than young men. However, we acknowledge the limitations of drawing inferences between the emerging literature in this area and the results of our study and suggest that further research is needed to confirm our speculations above.

There is longstanding debate in the literature about the age-period-cohort design as summarised by Bell and colleagues.^57^^,^^58^ This is due to the collinear relationship between age, period and cohort such that the value of one of these variables (e.g., age) can always be calculated from the values of the other two (e.g., period minus cohort). Standard regression models, such as entering all three variables as factors into a Poisson regression model, cannot estimate the effect sizes for all three variables reliably. A number of solutions have been proposed to solve this problem (e.g., constraining some parameters to be equal, creating course groupings for some variables and precise groupings for others, omitting one variable so that an age-period or age-cohort model is estimated instead) and these techniques are where much of the criticism about age-period-cohort models have been directed.^57^, ^58^, ^59^ The approach we have used instead has been developed relatively recently and models age, period and cohort effects as non-linear continuous variables using restricted cubic splines.^21^ To address the identifiability problem, the vectors for the splines representing the period and cohort effects are transformed to remove any underlying trend (i.e., to have a slope of 0 and average of 0 on the log scale) with a drift term then added for the cohort effect.^21^^,^^22^^,^^60^ This approach enables estimates of age, period and cohort effects to be interpreted individually and depicted graphically.^22^ Nonetheless, the method assumes that effects we are observing can be reasonably modelled as continuous non-linear variables and that the assumptions made to deal with the identifiably problem are appropriate.

Our study has both strengths and limitations. Strengths include a long period of data available for analysis, utilising data from people born in 1823 through to 2010. We were able to use contemporary epidemiological methods to identify different age, period and cohort effects and we were able to stratify by sex. Limitations include a reliance on date of death registration—as opposed to date of death—to anchor any period and cohort effects. In most case, these dates will be the same at the yearly level but there will be situations where they are not. The study relies on 10 different editions of the ICD to classify death by suicide. There may, therefore, be systematic differences in the coding of suicide over long time periods that may mean suicides are under- and over-reported at different times. We have modelled rates for the whole population with the only stratification by sex. Different patterns are likely to exist in Aboriginal and Torres Strait Islander groups, which have a much higher suicide rate than the rest of the Australian population,^1^ and different patterns may exist in some regions. Changes in suicide rates may relate to significant shifts in the social and cultural context (e.g., changes in gender equality). These shifts cannot easily be accounted for in an age-period-cohort study, mainly because on the unavailability of theoretically robust exposure data over a long-time frame. Since 2008 the Australian Bureau of Statistics has revised yearly counts of suicides to ensure that cases that had an undetermined cause of death when the mortality statistics were compiled can be counted as suicide if these cases were subsequently resolved and determined to be suicide. Thus, increases in suicides may in part be due to this process. To address the identifiably problem that is common in age, period and cohort studies, we modelled these effects as continuous non-linear variables. This removes the linear trend from the cohort and period effects but means that the resulting cohort or period curves are at risk of over-interpretation because the underlying linear effect is unknown. Our extrapolations about future suicide rates, especially for women, assume that the trends we have observed will continue in the future. Similarly, we assume there is no future external events that might increase or decrease the suicide rate. Finally, like all age-period-cohort studies, we have analysed aggregate-level data and are therefore at risk of the ecological fallacy. Patterns observed at the population level may not hold for individuals, and we do not know if individuals who died by suicide were exposed to the explanatory factors we have discussed (e.g., digital media consumption).

In summary, we found that changes in the suicide rates in Australia arise in the context of a complex interplay between age, cohort and period effects. For both males and females, each cohort born after 1956–1950 has a greater lifetime risk of suicide than the cohort born previously. For both sexes, the risk of suicide has additionally increased each year since the mid-late 2000s. If these trends continue, suicide rates could increase for women in all age groups but particularly young women. The impact of these factors on suicide rates in men is less clear.

## Contributors

MJS was responsible for the study concept and design, acquired the data and did the statistical analyses. All authors interpreted the data. MJS, RM, MS and AP drafted the manuscript. All authors revised and critically analysed the manuscript for important intellectual content. All authors had full access to the data used in the study. MJS had final responsibility for the decision to submit for publication.

## Data sharing statement

Data are available to download from the Australian Institute of Health and Welfare (https://www.aihw.gov.au/reports/life-expectancy-death/grim-books/contents/grim-books).

## Declaration of interests

Matthew Spittal holds a National Health and Medical Research Council Investigator Grant (GNT2025205) which supports his salary and research costs. Rachel Mitchell receives research salary support from the University of Toronto, Department of Psychiatry, and Sunnybrook Health Sciences Centre, Department of Psychiatry. Angela Clapperton holds a Suicide Prevention Australia Postdoctoral Fellowship which supports her salary. Mark Sinyor receives research salary support from the University of Toronto, Department of Psychiatry, and Sunnybrook Health Sciences Centre, Department of Psychiatry.

## References

[1] Australian Bureau of Statistics (2021).

[2] Stefanac N., Hetrick S., Hulbert C., Spittal M.J., Witt K., Robinson J. (2019). Are young female suicides increasing? A comparison of sex-specific rates and characteristics of youth suicides in Australia over 2004-2014. BMC Public Health.

[3] Lahti A., Räsänen P., Riala K., Keränen S., Hakko H. (2011). Youth suicide trends in Finland, 1969–2008. J Child Psychol Psychiatry.

[4] Skinner R., McFaull S. (2012). Suicide among children and adolescents in Canada: trends and sex differences, 1980–2008. CMAJ.

[5] Sullivan E.M., Annest J.L., Simon T.R., Luo F., Dahlberg L.L. (2015). Suicide trends among persons aged 10–24 years—United States, 1994–2012. Morb Mortal Wkly Rep.

[6] Mitchell R.H.B., Kozloff N., Sanches M. (2023). Sex differences in suicide trends among adolescents aged 10 to 14 Years in Canada. Can J Psychiatr.

[7] Botha F., Morris R.W., Butterworth P., Glozier N. (2023). Generational differences in mental health trends in the twenty-first century. Proc Natl Acad Sci USA.

[8] Morrell S., Page A., Taylor R. (2002). Birth cohort effects in New South Wales suicide, 1865–1998. Acta Psychiatr Scand.

[9] Page A., Milner A., Morrell S., Taylor R. (2013). The role of under-employment and unemployment in recent birth cohort effects in Australian suicide. Soc Sci Med.

[10] Oliver R., Hetzel B. (1972). Rise and fall of suicide rates in Australia: relation to sedative availability. Med J Aust.

[11] Morrell S., Taylor R., Quine S., Kerr C. (1993). Suicide and unemployment in Australia 1907–1990. Soc Sci Med.

[12] Thomas K., Gunnell D. (2010). Suicide in England and Wales 1861–2007: a time-trends analysis. Int J Epidemiol.

[13] Chang S.-S., Gunnell D., Sterne J.A.C., Lu T.-H., Cheng A.T.A. (2009). Was the economic crisis 1997–1998 responsible for rising suicide rates in East/Southeast Asia? A time–trend analysis for Japan, Hong Kong, South Korea, Taiwan, Singapore and Thailand. Soc Sci Med.

[14] Chang S.-S., Stuckler D., Yip P., Gunnell D. (2013). Impact of 2008 global economic crisis on suicide: time trend study in 54 countries. BMJ.

[15] Reeves A., Stuckler D., McKee M., Gunnell D., Chang S.-S., Basu S. (2012). Increase in state suicide rates in the USA during economic recession. Lancet.

[16] Chen Y.-Y., Chen M., Lui C.S., Yip P.S. (2017). Female labour force participation and suicide rates in the world. Soc Sci Med.

[17] Yip P.S., Chen Y.-Y., Yousuf S. (2012). Towards a reassessment of the role of divorce in suicide outcomes: evidence from five pacific rim populations. Soc Sci Med.

[18] Rossow I. (1993). Suicide, alcohol, and divorce; aspects of gender and family integration. Addiction.

[19] Twenge J.M., Martin G.N. (2020). Gender differences in associations between digital media use and psychological well-being: evidence from three large datasets. J Adolesc.

[20] Australian Institute of Health and Welfare (2022). General records of incidence of mortality (GRIM) books. https://www.aihw.gov.au/reports/life-expectancy-death/grim-books/contents/grim-books.

[21] Carstensen B. (2007). Age–period–cohort models for the Lexis diagram. Stat Med.

[22] Dobson A., Hockey R., Chan H.-W., Mishra G. (2020). Flexible age-period-cohort modelling illustrated using obesity prevalence data. BMC Med Res Methodol.

[23] Berger-Thomson L., Breusch J., Lilley L., Treasury T. (2018). Australia’s experience with economic reform, Treasury Working Paper.

[24] Rodrigues WTdS., Simões T.C., Magnago C. (2023). The influence of the age-period-cohort effects on male suicide in Brazil from 1980 to 2019. PLoS One.

[25] Thibodeau L. (2015). Suicide mortality in Canada and Quebec, 1926-2008: an age-period-cohort analysis. Can Stud Popul.

[26] Wang Z., Wang J., Bao J., Gao X., Yu C., Xiang H. (2016). Temporal trends of suicide mortality in mainland China: results from the age-periodperiod-cohort framework. Int J Environ Res Publ Health.

[27] Gunnell D., Middleton N., Whitley E., Dorling D., Frankel S. (2003). Influence of cohort effects on patterns of suicide in England and Wales, 1950–1999. Br J Psychiatr.

[28] Chen Y.-Y., Yang C.-T., Pinkney E., Yip P.S. (2021). The Age-Period-Cohort trends of suicide in Hong Kong and Taiwan, 1979-2018. J Affect Disord.

[29] Kino S., Jang S.-N., Gero K., Kato S., Kawachi I. (2019). Age, period, cohort trends of suicide in Japan and Korea (1986–2015): a tale of two countries. Soc Sci Med.

[30] Jukkala T., Stickley A., Mäkinen I.H., Baburin A., Sparén P. (2017). Age, period and cohort effects on suicide mortality in Russia, 1956− 2005. BMC Public Health.

[31] Park C., Jee Y.H., Jung K.J. (2016). Age–period–cohort analysis of the suicide rate in Korea. J Affect Disord.

[32] Granizo J.J., Guallar E., Rodriguez-Artalejo F. (1996). Age-period-cohort analysis of suicide mortality rates in Spain, 1959–1991. Int J Epidemiol.

[33] Ajdacic–Gross V., Bopp M., Gostynski M., Lauber C., Gutzwiller F., Rössler W. (2006). Age–period–cohort analysis of Swiss suicide data, 1881–2000. Eur Arch Psychiatr Clin Neurosci.

[34] Phillips J.A. (2014). A changing epidemiology of suicide? The influence of birth cohorts on suicide rates in the United States. Soc Sci Med.

[35] Friedland D.S., Price R.H. (2003). Underemployment: consequences for the health and well-being of workers. Am J Commun Psychol.

[36] Dooley D., Prause J., Ham-Rowbottom K.A. (2000). Underemployment and depression: longitudinal relationships. J Health Soc Behav.

[37] Milner A., Page A., LaMontagne A.D. (2014). Cause and effect in studies on unemployment, mental health and suicide: a meta-analytic and conceptual review. Psychol Med.

[38] Orlins E., DeBois K., Chatfield S.L. (2021). Characteristics of interpersonal conflicts preceding youth suicide: analysis of data from the 2017 National Violent Death Reporting System. Child Adolesc Ment Health.

[39] Evans R., Scourfield J., Moore G. (2016). Gender, relationship breakdown, and suicide risk: a review of research in Western countries. J Fam Issues.

[40] Wilcox H.C., Conner K.R., Caine E.D. (2004). Association of alcohol and drug use disorders and completed suicide: an empirical review of cohort studies. Drug Alcohol Depend.

[41] Lawrence D., Johnson S., Hafekost J. (2015).

[42] Atkinson G., Stanwick J. (2016).

[43] Bastiampillai T., Allison S., Looi J.C., Tavella A., Agis U. (2020). Why are Australia’s suicide rates returning to the hundred-year average, despite suicide prevention initiatives? Reframing the problem from the perspective of Durkheim. Aust N Z J Psychiatr.

[44] Taylor R., Lewis M., Powles J. (1998). The Australian mortality decline: all-cause mortality 1788-1990. Aust N Z J Publ Health.

[45] Page A., Spittal M.J. (2022). A decline in Australian suicide during COVID-19? A reflection on the 2020 cause of death statistics in the context of long-term trends. J Affect Disord Rep.

[46] Milner A., Morrell S., LaMontagne A.D. (2014). Economically inactive, unemployed and employed suicides in Australia by age and sex over a 10-year period: what was the impact of the 2007 economic recession?. Int J Epidemiol.

[47] Twenge J.M., Joiner T.E., Rogers M.L., Martin G.N. (2018). Increases in depressive symptoms, suicide-related outcomes, and suicide rates among US adolescents after 2010 and links to increased new media screen time. Clin Psychol Sci.

[48] Li H., Zhang X., Cao Y., Zhang G. (2023). Potential protection of computer gaming against mental health issues: evidence from a Mendelian randomization study. Comput Hum Behav.

[49] Ferguson C.J., Coulson M., Barnett J. (2011). A meta-analysis of pathological gaming prevalence and comorbidity with mental health, academic and social problems. J Psychiatr Res.

[50] Seabrook E.M., Kern M.L., Rickard N.S. (2016). Social networking sites, depression, and anxiety: a systematic review. JMIR Mental Health.

[51] Abi-Jaoude E., Naylor K.T., Pignatiello A. (2020). Smartphones, social media use and youth mental health. CMAJ.

[52] Marchant A., Hawton K., Stewart A. (2017). A systematic review of the relationship between internet use, self-harm and suicidal behaviour in young people: the good, the bad and the unknown. PLoS One.

[53] Abrutyn S., Mueller A.S. (2014). Are suicidal behaviors contagious in adolescence? Using longitudinal data to examine suicide suggestion. Am Socio Rev.

[54] Niederkrotenthaler T., Stack S., Till B. (2019). Association of increased youth suicides in the United States with the release of 13 Reasons Why. JAMA Psychiatr.

[55] Reidenberg D., Niederkrotenthaler T., Sinyor M., Bridge J.A., Till B. (2020). 13 Reasons Why: the evidence is in and cannot Be ignored. J Am Acad Child Adolesc Psychiatry.

[56] Sinyor M., Mallia E., de Oliveira C. (2021). Emergency department visits for self-harm in adolescents after release of the Netflix series ‘13 Reasons Why’. Aust N Z J Psychiatr.

[57] Bell A., Jones K. (2013). The impossibility of separating age, period and cohort effects. Soc Sci Med.

[58] Jones P.M., Minton J., Bell A. (2023). Methods for disentangling period and cohort changes in mortality risk over the twentieth century: comparing graphical and modelling approaches. Qual Quantity.

[59] Bell A. (2020). Age period cohort analysis: a review of what we should and shouldn’t do. Ann Hum Biol.

[60] Rutherford M.J., Lambert P.C., Thompson J.R. (2010). Age–period–cohort modeling. STATA J.

