# Supplementary material for: Age, period and cohort analysis of suicide trends in Australia, 1907–2020
Source: Lancet Reg Health West Pac. 2024 Aug 13;51:101171. doi: 10.1016/j.lanwpc.2024.101171 (PMC11379668; doi:10.1016/j.lanwpc.2024.101171)
Supplement: Supplementary Figs. S1 and S2 and Tables S1–S4 [file mmc1.docx]

# Age, period and cohort analysis of suicide trends in Australia, 1907 to 2020: Supplementary information

[Table S1: Goodness of fit statistics, males 2](#_Toc156907879)

[Table S2: Model coefficients for final age, period and cohort model, males 3](#_Toc156907880)

[Table S3: Goodness of fit statistics, females 4](#_Toc156907881)

[Table S4: Model coefficients for final age, period and cohort model, females 5](#_Toc156907882)

[Figure S1: Observed and fitted rates for selected birth cohorts, males 6](#_Toc156907883)

[Figure S2: Observed and fitted rates for selected birth cohorts, females 7](#_Toc156907884)

## Table S1: Goodness of fit statistics, males

| **Model** | **Cohort, number of knots** | **Period, number of knots** | **Log likelihood** | **Degrees of freedom** | **AIC** | **BIC** |
| --- | --- | --- | --- | --- | --- | --- |
| ACP | 5 | 5 | -8,912.56 | 15 | 17,855.12 | 17,936.78 |
| ACP | 5 | 6 | -8,784.76 | 16 | 17,601.53 | 17,688.63 |
| ACP | 5 | 7 | -8,741.75 | 17 | 17,517.51 | 17,610.06 |
| ACP | 5 | 8 | -8,358.50 | 18 | 16,753.01 | 16,851.00 |
| ACP | 5 | 9 | -8,307.12 | 19 | 16,652.23 | 16,755.67 |
| ACP | 6 | 5 | -8,738.59 | 16 | 17,509.18 | 17,596.29 |
| ACP | 6 | 6 | -8,609.31 | 17 | 17,252.62 | 17,345.17 |
| ACP | 6 | 7 | -8,566.56 | 18 | 17,169.13 | 17,267.13 |
| ACP | 6 | 8 | -8,181.72 | 19 | 16,401.44 | 16,504.88 |
| ACP | 6 | 9 | -8,129.16 | 20 | 16,298.32 | 16,407.21 |
| ACP | 7 | 5 | -8,656.09 | 17 | 17,346.17 | 17,438.73 |
| ACP | 7 | 6 | -8,530.70 | 18 | 17,097.40 | 17,195.40 |
| ACP | 7 | 7 | -8,486.91 | 19 | 17,011.82 | 17,115.26 |
| ACP | 7 | 8 | -8,102.95 | 20 | 16,245.89 | 16,354.78 |
| **ACP** | **7** | **9** | **-8,050.88** | **21** | **16,143.76** | **16,258.09** |
| ACP | 8 | 5 | -8,661.85 | 18 | 17,359.70 | 17,457.69 |
| ACP | 8 | 6 | -8,540.99 | 19 | 17,119.98 | 17,223.42 |
| ACP | 8 | 7 | -8,490.05 | 20 | 17,020.10 | 17,128.99 |
| ACP | 8 | 8 | -8,111.99 | 21 | 16,265.98 | 16,380.31 |
| ACP | 8 | 9 | -8,059.20 | 22 | 16,162.40 | 16,282.18 |
| ACP | 9 | 5 | -8,680.43 | 19 | 17,398.86 | 17,502.30 |
| ACP | 9 | 6 | -8,557.99 | 20 | 17,155.98 | 17,264.86 |
| ACP | 9 | 7 | -8,507.78 | 21 | 17,057.56 | 17,171.89 |
| ACP | 9 | 8 | -8,125.40 | 22 | 16,294.81 | 16,414.58 |
| ACP | 9 | 9 | -8,073.26 | 23 | 16,192.52 | 16,317.74 |
| AC | 5 | - | -10501.60 | 11 | 21,025.20 | 21,085.08 |
| AC | 6 | - | -10266.21 | 12 | 20,556.42 | 20,621.75 |
| AC | 7 | - | -10188.29 | 13 | 20,402.57 | 20,473.35 |
| AC | 8 | - | -10201.93 | 14 | 20,431.86 | 20,508.08 |
| AC | 9 | - | -10217.59 | 15 | 20,465.18 | 20,546.84 |
| AP | - | 5 | -12111.42 | 11 | 24,244.84 | 24,304.72 |
| AP | - | 6 | -12010.50 | 12 | 24,045.00 | 24,110.34 |
| AP | - | 7 | -11944.22 | 13 | 23,914.43 | 23,985.21 |
| AP | - | 8 | -11567.53 | 14 | 23,163.06 | 23,239.28 |
| AP | - | 9 | -11520.99 | 15 | 23,071.99 | 23,153.65 |

Notes. ACP = age-specific rates for each cohort after adjustment for period effects, AC = age-specific rates for each cohort, AP = age-specific rates for each period. AIC = Akaike information criterion; BIC = Bayesian information criterion. The model with the lowest AIC and BIC values is highlighted in bold.

## Table S2: Model coefficients for final age, period and cohort model, males

| **Variable** | **Coefficient** | **95% CI** | **p-value** |
| --- | --- | --- | --- |
| Age spline, knot 1 (intercept) | -8.5878 | (-8.6067 to -8.5688) | 0.0000 |
| Age spline, knot 2 | 0.4804 | (0.4702 to 0.4905) | 0.0000 |
| Age spline, knot 3 | 0.4066 | (0.3977 to 0.4155) | 0.0000 |
| Age spline, knot 4 | -0.2280 | (-0.2350 to -0.2210) | 0.0000 |
| Age spline, knot 5 | 0.0423 | (0.0361 to 0.0485) | 0.0000 |
| Age spline, knot 6 | -0.0687 | (-0.0753 to -0.0620) | 0.0000 |
| Period spline, knot 1 | 0.1407 | (0.1316 to 0.1498) | 0.0000 |
| Period spline, knot 2 | 0.1022 | (0.0949 to 0.1095) | 0.0000 |
| Period spline, knot 3 | 0.0883 | (0.0804 to 0.0961) | 0.0000 |
| Period spline, knot 4 | 0.0749 | (0.0666 to 0.0831) | 0.0000 |
| Period spline, knot 5 | -0.0520 | (-0.0563 to -0.0477) | 0.0000 |
| Period spline, knot 6 | 0.0252 | (0.0196 to 0.0308) | 0.0000 |
| Period spline, knot 7 | -0.0067 | (-0.0116 to -0.0019) | 0.0068 |
| Period spline, knot 8 | -0.0559 | (-0.0624 to -0.0494) | 0.0000 |
| Cohort spline, knot 1 (drift) | -0.0002 | (-0.0004 to -0.0000) | 0.0173 |
| Cohort spline, knot 2 | -0.2808 | (-0.2932 to -0.2685) | 0.0000 |
| Cohort spline, knot 3 | 0.0675 | (0.0623 to 0.0727) | 0.0000 |
| Cohort spline, knot 4 | 0.0352 | (0.0294 to 0.0411) | 0.0000 |
| Cohort spline, knot 5 | 0.0162 | (0.0096 to 0.0229) | 0.0000 |
| Cohort spline, knot 6 | -0.0204 | (-0.0285 to -0.0124) | 0.0000 |
| Cohort spline, knot 7 | -0.1656 | (-0.1769 to -0.1543) | 0.0000 |

## Table S3: Goodness of fit statistics, females

| **Model** | **Cohort, number of knots** | **Period, number of knots** | **Log likelihood** | **Degrees of freedom** | **AIC** | **BIC** |
| --- | --- | --- | --- | --- | --- | --- |
| ACP | 5 | 5 | -5,700.15 | 15 | 11,430.31 | 11,511.97 |
| ACP | 5 | 6 | -5,543.20 | 16 | 11,118.40 | 11,205.51 |
| ACP | 5 | 7 | -5,595.77 | 17 | 11,225.54 | 11,318.09 |
| ACP | 5 | 8 | -5,455.20 | 18 | 10,946.40 | 11,044.40 |
| ACP | 5 | 9 | -5,478.96 | 19 | 10,995.91 | 11,099.35 |
| ACP | 6 | 5 | -5,695.01 | 16 | 11,422.03 | 11,509.14 |
| ACP | 6 | 6 | -5,538.86 | 17 | 11,111.72 | 11,204.27 |
| ACP | 6 | 7 | -5,591.36 | 18 | 11,218.72 | 11,316.72 |
| ACP | 6 | 8 | -5,450.41 | 19 | 10,938.83 | 11,042.27 |
| ACP | 6 | 9 | -5,474.11 | 20 | 10,988.22 | 11,097.10 |
| ACP | 7 | 5 | -5,690.85 | 17 | 11,415.69 | 11,508.24 |
| ACP | 7 | 6 | -5,536.18 | 18 | 11,108.37 | 11,206.37 |
| ACP | 7 | 7 | -5,588.34 | 19 | 11,214.69 | 11,318.13 |
| **ACP** | **7** | **8** | **-5,447.08** | **20** | **10,934.16** | **11,043.04** |
| ACP | 7 | 9 | -5,470.72 | 21 | 10,983.45 | 11,097.78 |
| ACP | 8 | 5 | -5,690.80 | 18 | 11,417.61 | 11,515.61 |
| ACP | 8 | 6 | -5,537.37 | 19 | 11,112.75 | 11,216.19 |
| ACP | 8 | 7 | -5,589.33 | 20 | 11,218.65 | 11,327.54 |
| ACP | 8 | 8 | -5,448.54 | 21 | 10,939.09 | 11,053.42 |
| ACP | 8 | 9 | -5,472.06 | 22 | 10,988.12 | 11,107.90 |
| ACP | 9 | 5 | -5,692.43 | 19 | 11,422.86 | 11,526.30 |
| ACP | 9 | 6 | -5,538.17 | 20 | 11,116.34 | 11,225.22 |
| ACP | 9 | 7 | -5,590.45 | 21 | 11,222.91 | 11,337.24 |
| ACP | 9 | 8 | -5,448.80 | 22 | 10,941.59 | 11,061.37 |
| ACP | 9 | 9 | -5,472.74 | 23 | 10,991.48 | 11,116.70 |
| AC | 5 | - | -6,539.08 | 11 | 13,100.15 | 13,160.04 |
| AC | 6 | - | -6,535.19 | 12 | 13,094.37 | 13,159.70 |
| AC | 7 | - | -6,532.54 | 13 | 13,091.08 | 13,161.86 |
| AC | 8 | - | -6,532.61 | 14 | 13,093.23 | 13,169.45 |
| AC | 9 | - | -6,533.41 | 15 | 13,096.82 | 13,178.48 |
| AP | - | 5 | -6,199.77 | 11 | 12,421.54 | 12,481.42 |
| AP | - | 6 | -6,039.92 | 12 | 12,103.83 | 12,169.16 |
| AP | - | 7 | -6,095.93 | 13 | 12,217.87 | 12,288.64 |
| AP | - | 8 | -5,951.19 | 14 | 11,930.38 | 12,006.60 |
| AP | - | 9 | -5,975.75 | 15 | 11,981.51 | 12,063.17 |

Notes. ACP = age-specific rates for each cohort after adjustment for period effects, AC = age-specific rates for each cohort, AP = age-specific rates for each period. AIC = Akaike information criterion; BIC = Bayesian information criterion. The model with the lowest AIC and BIC values is highlighted in bold.

## Table S4: Model coefficients for final age, period and cohort model, females

|  | **Coefficient** | **95% CI** | **p-value** |
| --- | --- | --- | --- |
| Age spline, knot 1 (intercept) | -9.7672 | (-9.7996 to -9.7348) | 0.0000 |
| Age spline, knot 2 | 0.3243 | (0.3071 to 0.3414) | 0.0000 |
| Age spline, knot 3 | 0.4826 | (0.4672 to 0.4980) | 0.0000 |
| Age spline, knot 4 | -0.1346 | (-0.1469 to -0.1222) | 0.0000 |
| Age spline, knot 5 | 0.0420 | (0.0310 to 0.0529) | 0.0000 |
| Age spline, knot 6 | -0.0692 | (-0.0809 to -0.0576) | 0.0000 |
| Period spline, knot 1 | 0.1250 | (0.1079 to 0.1421) | 0.0000 |
| Period spline, knot 2 | 0.1436 | (0.1272 to 0.1600) | 0.0000 |
| Period spline, knot 3 | -0.1537 | (-0.1635 to -0.1439) | 0.0000 |
| Period spline, knot 4 | 0.0945 | (0.0799 to 0.1092) | 0.0000 |
| Period spline, knot 5 | 0.0155 | (0.0068 to 0.0242) | 0.0005 |
| Period spline, knot 6 | 0.0254 | (0.0131 to 0.0377) | 0.0000 |
| Period spline, knot 7 | -0.0217 | (-0.0297 to -0.0137) | 0.0000 |
| Cohort spline, knot 1 (drift) | -0.0012 | (-0.0016 to -0.0009) | 0.0000 |
| Cohort spline, knot 2 | -0.0125 | (-0.0451 to 0.0202) | 0.4552 |
| Cohort spline, knot 3 | 0.0166 | (0.0076 to 0.0257) | 0.0003 |
| Cohort spline, knot 4 | -0.0130 | (-0.0227 to -0.0034) | 0.0081 |
| Cohort spline, knot 5 | -0.1390 | (-0.1691 to -0.1089) | 0.0000 |
| Cohort spline, knot 6 | -0.1149 | (-0.1311 to -0.0986) | 0.0000 |
| Cohort spline, knot 7 | -0.0559 | (-0.0672 to -0.0447) | 0.0000 |

## Figure S1: Observed and fitted rates for selected birth cohorts, males

## Figure S2: Observed and fitted rates for selected birth cohorts, females
